# Reinforcement of Cathode Interface Using a Dipolar Small Molecule for Enhancing Operational Stability of Perovskite Solar Cells

**DOI:** 10.1002/advs.202524338

**Published:** 2026-03-18

**Authors:** Dong Hyun Lee, Seok Woo Lee, Min Jun Choi, Ramesh Kumar Chitumalla, Sang Eun Yoon, Gyeong G. Jeon, Juan Anthony Prayogo, Dong Won Kim, Joonkyung Jang, Jinhee Heo, Dong Wook Chang, Jong H. Kim

**Affiliations:** ^1^ Department of Molecular Science Technology Ajou University Suwon Republic of Korea; ^2^ Department of Energy and Chemical Materials Engineering and CECS Core Research Institute Pukyong National University Busan Republic of Korea; ^3^ Department of Nanoenergy Engineering Pusan National University Busan Republic of Korea; ^4^ AI‐Superconvergence KIURI Translational Research Center Ajou University Suwon Republic of Korea; ^5^ Department of Materials Analysis Korea Institute of Materials Science (KIMS) Changwon Republic of Korea

**Keywords:** cathode interlayer, device stability, electrode corrosion, organic small molecule, perovskite solar cells

## Abstract

Perovskite solar cells (PSCs) with inverted structures have garnered considerable attention in photovoltaic research due to their high scalability and efficiency. However, challenges at the interface between the electron transport layer (ETL) and cathode, including energy level mismatch, halide ion migration, and metal degradation, limit performance and stability. In this study, we introduce a quinoxaline‐based high‐dipole material (MQPPO) as a novel cathode interlayer (CIL) for p‐i‐n structures. MQPPO improves energy level alignment, charge extraction, and interfacial stability through its strong dipole moment and hydrophobic properties. The superior performance of MQPPO‐incorporated devices was reflected in their power conversion efficiency (PCE), achieving 21.65% under 1 sun illumination. Additionally, MQPPO‐based PSCs demonstrated exceptional long‐term stability, maintaining over 80% of their initial efficiency after 1440 h. These results demonstrate that MQPPO is an effective CIL material for high‐performance and stable PSCs, advancing their potential for sustainable energy technologies.

## Graphical abstract

We report a high‐dipole interlayer, MQPPO, that simultaneously improves efficiency and stability in perovskite solar cells. Its strong interfacial dipole enhances photovoltaic performance, while its uniform coverage and strong interaction with Ag effectively suppress AgI formation, ensuring long‐term stability under moisture and thermal stress. This multifunctional approach provides an effective strategy for interface engineering.

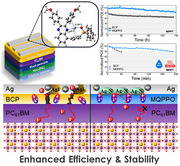

## 1 Introduction

Organic–inorganic hybrid perovskites have rapidly emerged as a high‐performance photovoltaic (PV) material due to their exceptional optoelectronic characteristics, including high absorption coefficient, tunable bandgap, long carrier diffusion length, and high charge mobility [1, 2, 3, 4, 5]. The power conversion efficiency (PCE) of single‐junction perovskite solar cells (PSCs) has risen dramatically from 3.8% to 27.0%, highlighting their potential to compete and even surpass conventional silicon‐based PV technologies [6, 7]. Among the various device configurations, the inverted p‐i‐n structure has attracted considerable attention because of its low‐temperature processability and scalability, particularly in tandem solar cells [8, 9, 10, 11]. Unlike the conventional n‐i‐p structure, which often employs doped hole transport layers (HTLs) such as 2,2’,7,7’‐tetrakis[N,N‐di(4‐methoxyphenyl)amino]‐9,9’‐spirobifluorene (Spiro‐OMeTAD), inverted PSCs eliminate the use of hygroscopic charge transport layers that are susceptible to degradation, thereby improving their long‐term operational stability [12].

Despite these advantages, inverted PSCs still face critical interfacial challenges at the electron transport layer (ETL)/cathode junction that limit both efficiency and durability. The energy level mismatch between phenyl‐C61‐butyric acid methyl ester (PC_61_BM), a widely used ETL material, and the Ag cathode results in charge accumulation and interfacial recombination losses [13, 14]. Furthermore, halide ion diffusion through the ETL and subsequent reaction with Ag forms insulating AgI, severely compromising PCE and device stability [15, 16]. To mitigate such interfacial deterioration, cathode interlayers (CILs) have been studied to optimize energy alignment, facilitate electron extraction, and block ion migration [17]. Bathocuproine (BCP) is one of the most commonly used CILs owing to its simple processing and dipole‐tuning capabilities [18, 19]. However, its tendency to aggregate often results in incomplete coverage of the ETL, insufficient ion blocking, and metal corrosion, which collectively degrade long‐term device performance [20]. Consequently, alternative materials, such as small organic molecules [21, 22], polymers [23], and metal oxides [24], have been investigated. These materials not only enhance charge extraction but also form uniform layers that improve operational stability. However, most reported CILs focus primarily on passivation, and only a few exhibit chemical interactions capable of suppressing interfacial reactions between halide ions and metal electrodes [25, 26].

To address these limitations, here, we introduce a quinoxaline‐based dipolar small molecule (4‐(2,3‐bis(4‐methoxyphenyl)quinoxalin‐5‐yl)phenyl)diphenylphosphine oxide (MQPPO), as an effective CIL in inverted PSCs [27]. MQPPO establishes favorable interfacial energy alignment, promoting efficient charge extraction and reducing interfacial recombination, resulting in a high PCE of 21.65% from p‐i‐n PSCs. Moreover, MQPPO forms uniform, pinhole‐free interfacial films and exhibits stronger binding affinity toward Ag than BCP, effectively suppressing halide diffusion and AgI formation. As a result, the devices retain over 80% of their initial PCE after 1440 h under ambient conditions, underscoring the excellent long‐term stability enabled by the MQPPO CIL.

## 2 Results and Discussion

**FIGURE 1:**
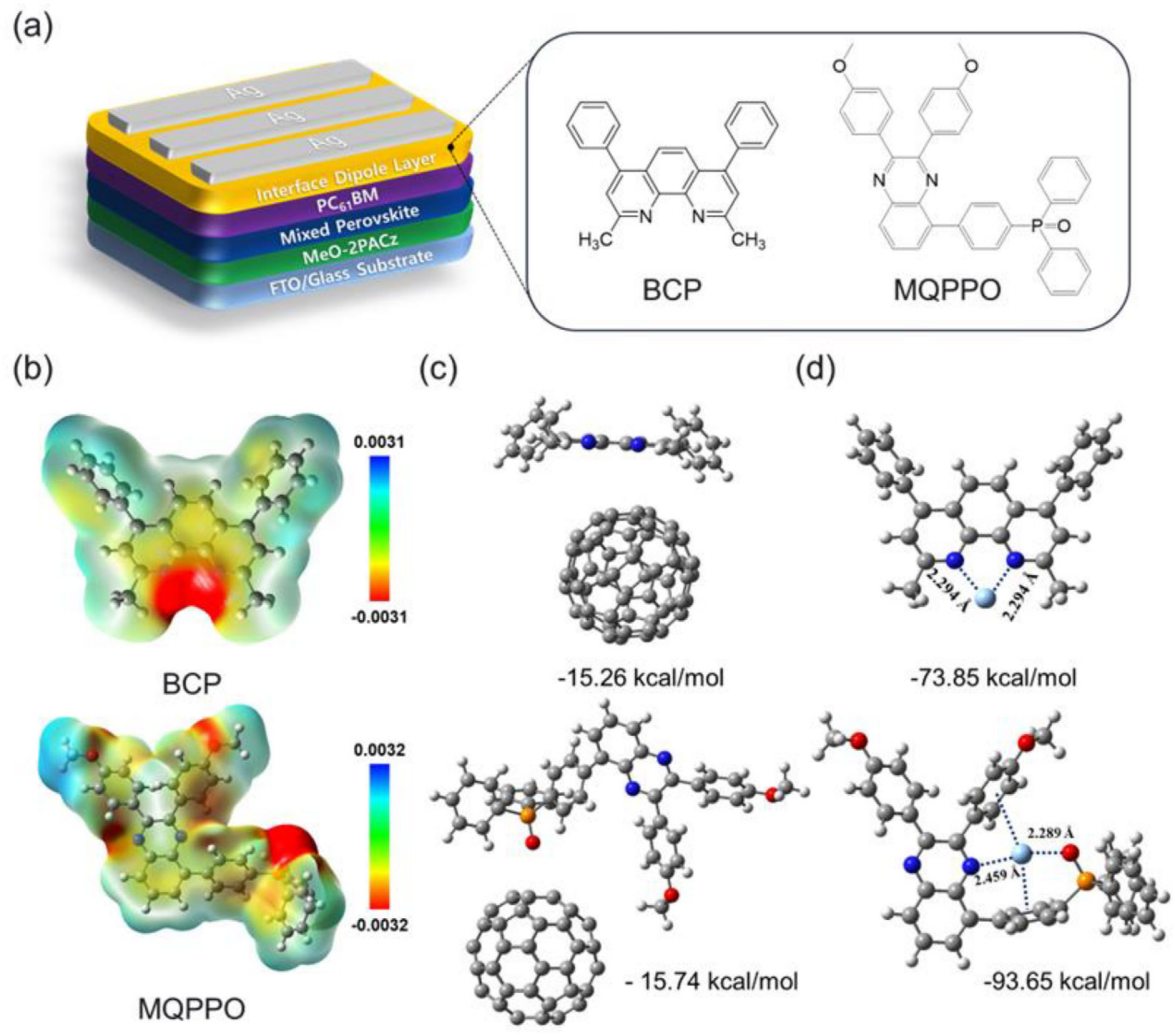
(a) Device architecture and chemical structures of cathode interlayers (BCP and MQPPO). (b) Simulated electrostatic potential (ESP) maps of BCP and MQPPO, with potential values shown on a color scale in electron volts (eV). (c,d) Molecular interactions of CILs with C_60_ and Ag, showing the optimized structures, calculated interaction energies, and corresponding interaction distances. Atoms are color‐coded as follows: carbon (gray), oxygen (red), phosphorus (orange), nitrogen (blue), and hydrogen (white).

In this study, a quinoxaline‐based dipolar molecule, MQPPO, was employed as the CIL in inverted p‐i‐n PSCs, replacing the conventional BCP used as the control. The devices were fabricated with an FTO/MeO‐2PACz/perovskite/PC_61_BM/CIL/Ag architecture, where MQPPO or BCP was inserted between PC_61_BM and Ag as CILs (Figure 1a). To elucidate the interfacial role of MQPPO, we performed density functional theory (DFT) calculations and electrostatic potential surface (ESP) analyses. As shown in Figure 1b, MQPPO exhibits a highly polarized dipole distribution compared to BCP. The ESP mapping reveals particularly stronger electron‐rich and electron‐deficient regions around the phosphine oxide moiety, indicating a larger dipole moment. Such a pronounced dipole is expected to facilitate favorable interfacial energy level alignment and enhance charge extraction across the PC_61_BM/Ag interface [28, 29].

Molecular interaction simulations were conducted using C_60_, a representative model for PC_61_BM, to evaluate interfacial binding affinity (Figure 1c). MQPPO showed a stronger binding interaction with C_60_ (−15.74 kcal/mol) than BCP (−15.26 kcal/mol), suggesting enhanced molecular contact that can promote interfacial charge transfer [30]. To further assess the chemical passivation capability, interactions with Ag^+^ ions were examined (Figure 1d). MQPPO displayed a notably stronger binding energy of −93.65 kcal/mol through coordination between nitrogen and oxygen atoms in its quinoxaline and phosphine oxide moieties, compared to −73.85 kcal/mol for BCP. This strong interaction is expected to suppress Ag─I bond formation, thereby preventing electrode corrosion and enhancing interfacial stability in MQPPO‐based PSCs [31]. In addition, the electron reorganization energy (ERE) of both CILs was calculated based on Marcus theory [32]. MQPPO exhibited a lower ERE (0.41 eV) than BCP (0.43 eV), indicating a more favorable electronic configuration for charge transfer. The reduced ERE of MQPPO is therefore conducive to more efficient and faster electron transfer across the PC_61_BM/Ag interface [33].

To further elucidate the interaction between MQPPO and PC_61_BM, we performed Fourier‐transform infrared spectroscopy (FTIR) measurements for MQPPO, PC_61_BM, PC_61_BM/MQPPO mixture, and PC_61_BM/BCP mixture (Figure S1). From the PC_61_BM/MQPPO mixture, the characteristic stretching vibration of the ester (C═O) group in PC_61_BM exhibited a more noticeable shift (3.9 cm^−1^) than that of the PC_61_BM/BCP mixture (0.7 cm^−1^). These results indicate that the oxygen atom in the phosphine oxide (P═O) group of MQPPO, acting as a strong Lewis base site, forms a significant chemical interaction with the electron‐deficient fullerene core of PC_61_BM (a Lewis acid) [34, 35]. This chemical coordination not only improves the interfacial contact between the PC_61_BM and MQPPO layer but also effectively passivates interfacial trap sites [20].

We conducted ultraviolet photoelectron spectroscopy (UPS) to evaluate the work function (WF) of Ag electrodes modified with different CILs (Figure S2). The WFs of pristine Ag, Ag/BCP, and Ag/MQPPO were determined to be 4.45, 4.30, and 3.95 eV, respectively. Based on the LUMO level of PC_61_BM (3.84 eV), the electron extraction barrier (ΔEe = |Φ_Ag_—LUMO_PC61BM_|) was significantly reduced from 0.61 eV (Ag) to 0.11 eV (Ag/MQPPO). This shift facilitates Ohmic‐like contact, promoting efficient charge transfer at the interface (Figure S3) [36]. The larger dipole moment of MQPPO (7.46 D) compared to BCP (3.31 D) [27, 37] induces a more pronounced WF shift of Ag, promoting favorable energy level alignment and facilitating electron extraction at the interface [38, 39, 40]. Furthermore, the larger barrier (2.03 eV) between Ag and MQPPO confirms hole‐blocking capability, effectively suppressing interfacial recombination (Figure S3). Additionally, as shown in Figure S4 and Table S1, lower photoluminescence (PL) intensity and shorter average decay lifetime of perovskite on MQPPO (τ_ave_ = 56.32 ns) compared to that on BCP (τ_ave_ = 63.77 ns), indicate more efficient charge extraction at the interface and reduced non‐radiative carrier recombination [41].

**FIGURE 2:**
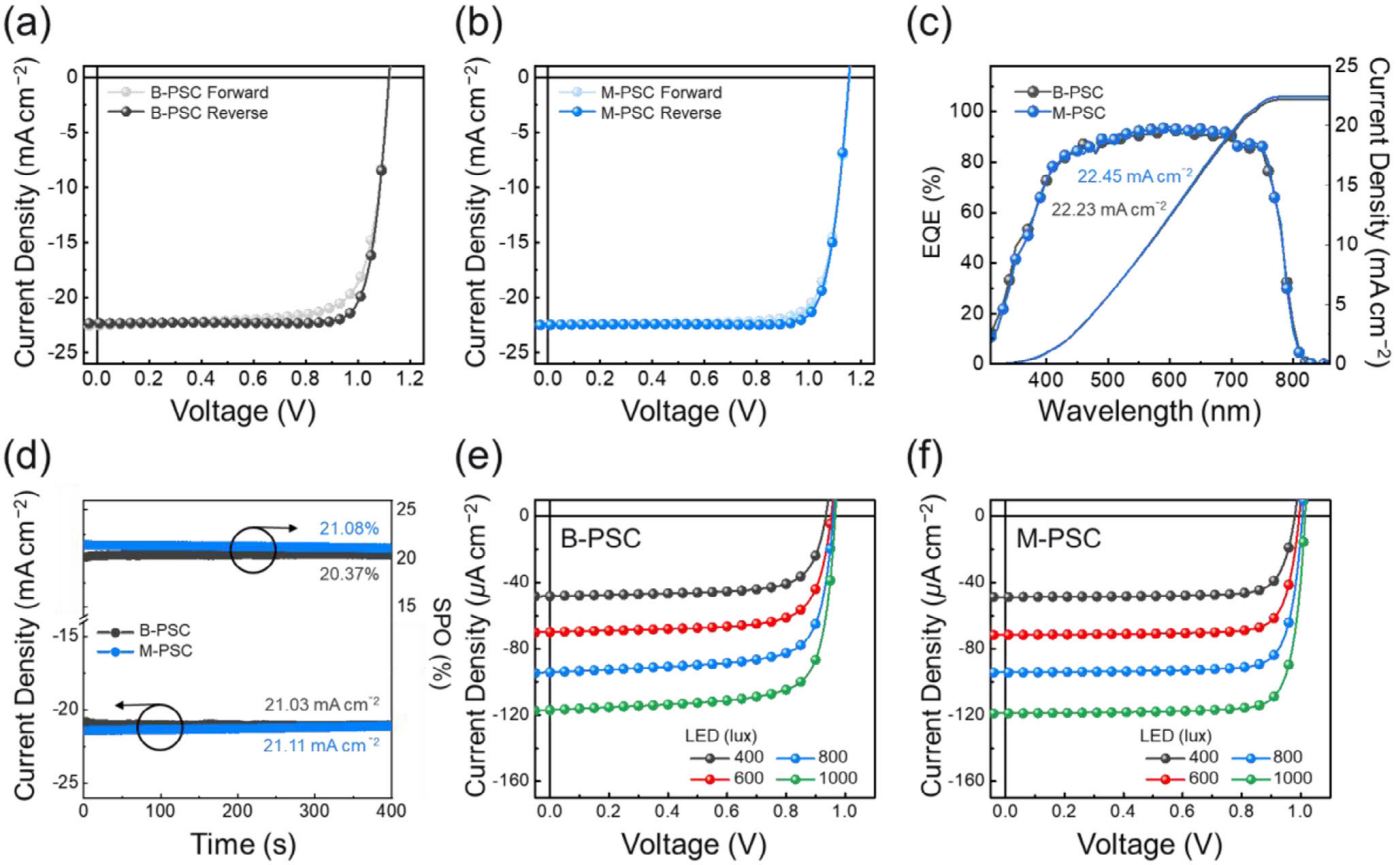
*J–V* curves for the (a) B‐PSC and (b) M‐PSC under AM1.5G 1‐sun illumination. (c) EQE spectra and (d) SPO measurement results of the B‐PSC and M‐PSC. *J–V* curves for the (e) B‐PSC and (f) M‐PSC under various LED illumination intensities (400, 600, 800, 1000 lux).

To investigate the dipole effect of MQPPO, we measured the current density (*J*)–voltage (*V*) characteristics under AM 1.5G 1 sun illumination. The dependence of the performances of M‐PSCs on MQPPO concentrations is summarized in Figure S5. The BCP‐based PSC (B‐PSC) exhibited a PCE of 20.73% with an open‐circuit voltage (*V*_OC_) of 1.12 V, short‐circuit current density (*J*_SC_) of 22.30 mA cm^−2^, and fill factor (FF) of 0.83 (Figure 2a). In contrast, the MQPPO‐based device (M‐PSC) achieved an enhanced PCE of 21.65% with a *V*_OC_ of 1.16 V, *J*_SC_ of 22.49 mA cm^−2^, and FF of 0.83 (Figure 2b). The average PV parameters are summarized in Table 1. Both *V*_OC_ and FF were improved for the M‐PSCs compared to those of B‐PSCs (Figure S6). Furthermore, the external quantum efficiency (EQE) spectra confirmed that the integrated *J*_SC_ values were consistent with those obtained from the *J–V* curves (Figure 2c). The stability of the PV output was validated via steady‐state power output (SPO) measurements (Figure 2d), confirming the reliability of the PV properties of both PSCs.

**TABLE 1:** Performance parameters of the PSCs using various CILs under 1‐sun illumination.

Devices^a^	Scan direction	*V*_OC_ (V)	*J*_SC_ (mA cm^−2^)	FF	PCE (%)
**B‐PSC**	FS	1.13	22.54	0.76	19.36
	RS	1.12 (1.13)	22.30 (22.28)	0.83 (0.81)	20.73 (20.25)
**M‐PSC**	FS	1.16	22.49	0.80	20.80
	RS	1.16 (1.15)	22.49 (22.48)	0.83 (0.82)	21.65 (21.25)

^a^ The average photovoltaic parameters of PSCs were obtained for 15 devices.

We also evaluated the *J–V* characteristics under varying indoor low‐light conditions (400, 600, 800, and 1000 lux) using the LED light sources (Figure 2e,f and Table 2). In indoor environments, where illumination levels are significantly lower than outdoor conditions, trap‐assisted recombination significantly affects device performance due to the limited photo‐generation of charge carriers [42]. Therefore, the incorporation of MQPPO was expected to suppress non‐radiative recombination while enabling efficient charge extraction through its strong interface dipole effect, preventing charges from being trapped in defects [43]. The indoor PCE (iPCE) of M‐PSCs increased from 25.91%, 25.87%, 26.61%, and 27.73% (for B‐PSCs) to 31.79%, 31.55%, 32.01%, and 33.18% at 400, 600, 800, and 1000 lux, respectively. Notably, the M‐PSCs exhibited an increased *V*_OC_ and FF compared to BCP.

**TABLE 2:** Performance parameters of the PSCs using various CILs subjected to varying LED illumination intensities (400, 600, 800, and 1000 lux).

Devices^a^	Indoor light	Light intensity (lux)	*V*_OC_ (V)	*J*_SC_ (*µA* cm^−2^)	FF	iPD (*µW* cm^−2^)	iPCE (%)
**B‐PSC**	LED	400	0.94 (0.91)	45.93 (46.84)	0.72 (0.69)	31.09 (29.49)	25.91 (24.58)
		600	0.96 (0.93)	68.67 (68.51)	0.71 (0.70)	46.81 (44.85)	25.87 (24.79)
		800	0.97 (0.95)	89.70 (90.11)	0.73 (0.72)	63.52 (61.31)	26.61 (25.68)
		1,000	1.00 (0.97)	117.16 (113.30)	0.71 (0.73)	83.18 (79.58)	27.73 (26.53)
**M‐PSC**	LED	400	0.99 (0.95)	48.78 (47.57)	0.79 (0.78)	38.15 (35.44)	31.79 (29.54)
		600	1.00 (0.97)	71.73 (70.21)	0.81 (0.79)	58.10 (53.95)	31.55 (29.82)
		800	1.01 (0.99)	94.34 (92.73)	0.81 (0.80)	77.18 (73.11)	32.01 (30.62)
		1,000	1.02 (1.00)	119.01 (116.87)	0.82 (0.81)	99.54 (94.66)	33.18 (31.55)

^a^ The average photovoltaic parameters of PSCs were measured in seven devices.

The high dipole moment of MQPPO effectively shifts the WF of the Ag electrode, an effect which is consequently expected to enhance electron extraction and suppress non‐radiative recombination [31]. To further investigate the recombination dynamics and charge transport, transient photocurrent (TPC), transient photovoltage (TPV), and photo‐CELIV measurements were performed. M‐PSCs showed faster photocurrent decay (1.35 µs) and longer photovoltage decay (3.88 µs) than B‐PSCs (2.03 µs and 2.73 µs, respectively), indicating enhanced charge extraction and suppressed recombination (Figure S7) [44]. Photo‐CELIV analysis revealed a comparable carrier mobility of 1.60 × 10^−3^ cm^2^ V^−1^ s^−1^ for M‐PSC and 1.57 × 10^−3^ cm^2^ V^−1^ s^−1^ for B‐PSC, confirming efficient charge transport at the ETL interface (Figure S8). For a deeper understanding of recombination, we characterized *J*_SC_ and *V*_OC_ depending on light intensity and determined *α* and *n* values using the relationship of *J*sc ∝ *I ^α^* and *V*_OC_ = (*nk*_B_ *T*/*q*)ln(*I*), where *α*, *I*, *n*, *k_B_*, *T*, and *q* are power factor, light intensity, ideality factor, Boltzmann constant, temperature, and elementary charge, respectively. Comparable *α* values close to 1.0 of both devices (0.98 and 0.97 for M‐PSC and B‐PSC, respectively) and the higher *n* value of the B‐PSC (1.68) compared to that of M‐PSC (1.48) imply that the observed recombination suppression in M‐PSC originated from effectively reduced trap‐assisted charge recombination by incorporating MQPPO (Figure S9) [45, 46, 47]. Furthermore, intensity modulated photocurrent spectroscopy (IMPS) and intensity modulated photovoltage spectroscopy (IMVS) analyses confirmed a faster charge transport time (τ_ct_) and slower recombination time (τ_rec_) for M‐PSCs (Figure S10). These results collectively support that delayed trap‐assisted recombination and facilitated charge extraction efficiency improved *V*_OC_ and FF in M‐PSCs under both 1 sun and low‐light intensity illuminations [48, 49, 50].

The versatility of MQPPO for different electrodes was evaluated using a gold (Au) cathode, which is typically unsuitable for electron extraction due to its deep work function (∼5.3 eV) [51]. As shown in Figure S11, the M‐PSC with an Au cathode achieved a superior PCE of 21.58% based on higher FF (0.80) and *V*_OC_ (1.15 V) compared to the B‐PSC (PCE: 20.25%, FF: 0.78, and *V*_OC_: 1.13 V) due to its high interface dipole. This confirms that MQPPO effectively tunes the energy levels even for high‐work‐function metals to form Ohmic‐like contact. Additionally, we fabricated p‐i‐n PSCs using a different perovskite composition (FA_0.825_Cs_0.175_Pb(Br_0.125_I_0.875_)_3_) via an anti‐solvent‐free vacuum crystallization method [52] (Figure S12a). As shown in Figure S12b and Table S2, the M‐PSC achieved a PCE up to 23.18% with a high *V*_OC_ of 1.17 V and FF of 0.82, which is higher than that of the B‐PSC (*V*_OC_: 1.10 V, FF: 0.80, and PCE: 21.31%). In Figure S12c), the EQE spectra yielded integrated *J*_SC_ values of 23.39 mA cm^−2^ for the M‐PSC and 23.35 mA cm^−2^ for the B‐PSC, which are in good agreement with the values obtained from the *J–V* measurements. These results clearly demonstrate that the MQPPO strategy is not limited to a specific efficiency range and serves as a versatile platform for achieving competitive performance in high‐efficiency p‐i‐n PSCs.

**FIGURE 3:**
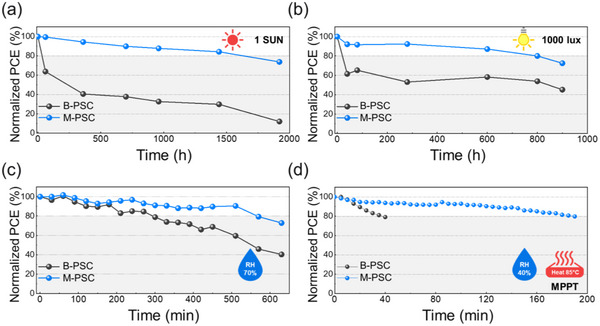
Ambient stability of the B‐PSC and M‐PSC without encapsulation under (a) 1‐sun and (b) 1000 lux LED illumination at 25°C/RH 40%. (c) Moisture stability of unencapsulated B‐PSC and M‐PSC was measured at 25°C/RH 70%. (d) MPPT measurement results for the B‐PSC and M‐PSC under accelerated thermal stress at 85 °C/RH 40%.

The long‐term stability of the unencapsulated devices was evaluated under various stress conditions to investigate the protective effect of the MQPPO interlayer. All aging experiments were performed in a temperature‐ and humidity‐controlled environmental chamber to ensure constant environmental conditions. Detailed operational procedures, including the specific environmental parameters for ambient, moisture, and accelerated thermal stability tests, are provided in Note S1. To examine the effect of the modified cathode interface on device stability, PSCs were stored under ambient conditions (25°C, relative humidity (RH) of 40%) without encapsulations, and we measured their PCE with time (Figure 3a,b). The B‐PSCs exhibited a rapid decrease in efficiency, retaining 80% of their initial PCE after 60 and 40 h under 1‐sun and 1000 LED lux illumination, respectively. In sharp contrast, the M‐PSCs maintained 80% of their initial performance even after 1440 h under 1‐sun illumination and 800 h under 1000 lux LED exposure, demonstrating superior ambient operational stability under versatile illumination conditions. In contrast to the substantial (45%) decrease in recombination resistance in B‐PSCs during stability tests, M‐PSCs exhibited a negligible change (0.4% decrease), suggesting the instability of the interfaces of B‐PSCs (Figure S13 and Table S3) [53].

To further evaluate moisture stability, we performed stability tests for unencapsulated devices under harsh humidity conditions (RH 70%) at 25°C. As shown in Figure 3c, the B‐PSCs showed severe degradation, retaining less than 40% of their initial efficiency after 600 min of storage. However, the M‐PSCs demonstrated much enhanced moisture resistance, maintaining over 70% of their initial PCE during the same period. Thermal stability was subsequently assessed under accelerated stress conditions by performing MPPT measurements at 85 °C (RH 40%) without encapsulation (Figure 3d). The B‐PSCs degraded within 40 min, while M‐PSCs maintained 80% of their initial efficiency for up to 190 min, demonstrating superior thermal and operational robustness. These results indicate that MQPPO provides enhanced resistance to both moisture and thermal degradation, ensuring more reliable operation under various environmental conditions [54].

**FIGURE 4:**
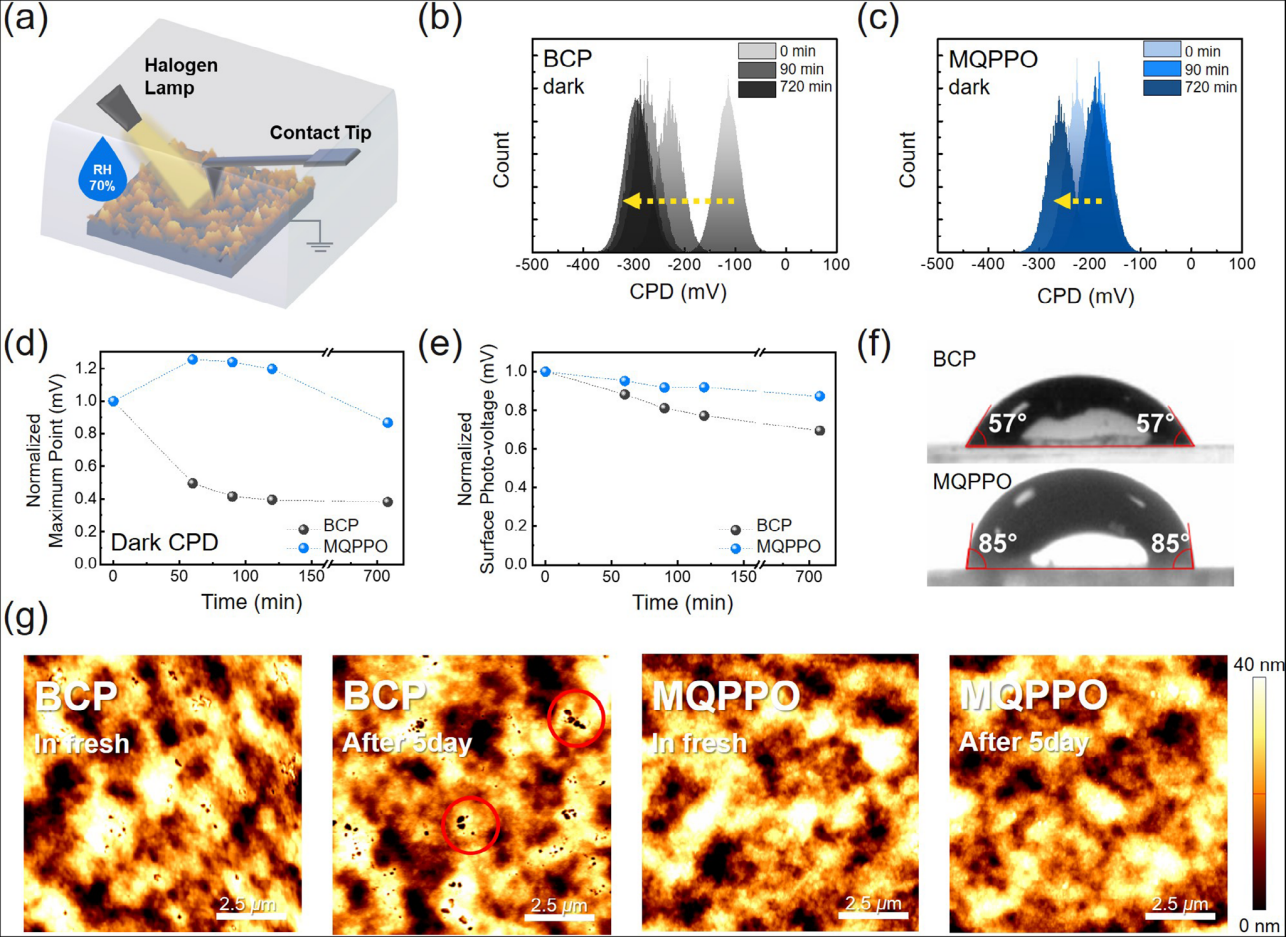
(a) Schematic illustration of the KPFM measurement under illumination at RH 70%. CPD distribution curves in dark conditions for perovskite films with (b) BCP and (c) MQPPO. Time‐dependent maximum (d) CPD values under dark conditions and (e) SPV values of the films with BCP and MQPPO. (f) Water contact angle measurements of the films based on BCP and MQPPO. (g) AFM topography images of the films with BCP and MQPPO in fresh and after 5‐days of storage in ambient conditions.

To elucidate the mechanisms underlying the enhanced stability of M‐PSCs through examination of changes in surface potential and interfacial electrical properties, we carried out Kelvin probe force microscopy (KPFM) measurements on perovskite/PC_61_BM/CIL (BCP or MQPPO) films (denoted as BCP‐based film or MQPPO‐based film) under dark and halogen lamp illumination environments at a RH 70% condition (Figure 4a) [55]. Through the measurements of time‐dependent dark contact potential difference (CPD_dark_) distributions (Figure 4b,c), we monitored the changes in their maximum peak positions for each film (Figure 4d). BCP‐based films exhibited a rapid decrease in peak position with time; however, the peak positions of MQPPO‐based films were well maintained. These results demonstrate that, in contrast to BCP, MQPPO maintains a strong interfacial dipole. Attributed to a robust interfacial dipole, the MQPPO‐based film showed much more stable surface photovoltage (SPV = CPD_light_−CPD_dark_, CPD_light_: CPD under illumination) with time, compared to that of the BCP‐based film, indicating stable and efficient charge extraction of photoexcited charge carriers. These results directly evidence improved operational stability of M‐PSCs under various environmental conditions (Figure 4e; Figure S14).

Additionally, as shown in Figure 4f, a larger contact angle (85°) of the MQPPO‐based film compared to the BCP‐based film (57°) demonstrates its superior hydrophobicity and improved resistance to moisture ingress. Furthermore, atomic force microscopy (AFM) was used to monitor the morphological changes by comparing fresh and 5‐day‐aged films under ambient conditions (25°C, RH 40%) (Figure 4g). Over time, the BCP‐based films developed pronounced dark spots, as a result of BCP aggregation, which fails to passivate the PC_61_BM surface and thereby leads to pinhole formation [56]. Specifically, such morphological non‐uniformity and pinholes in BCP films impair electrical contact and increase series resistance, leading to a decline in FF as previously reported [58, 59]. Furthermore, these structural defects serve as facilitated pathways for halide ion migration toward the Ag electrode, which accelerates electrode corrosion via AgI formation and ultimately undermines operational stability [60, 61, 62]. In contrast, MQPPO‐based films showed negligible morphological degradation, maintaining a uniform and compact surface even after five days. Additionally, x‐ray diffraction (XRD) analysis (Figure S15) revealed suppressed PbI_2_ formation in MQPPO‐based films, further evidencing reduced moisture‐induced decomposition of perovskite [63]. Collectively, these results suggest that MQPPO effectively reinforces interfacial stability by maintaining dipole effects, preventing moisture penetration, and suppressing interfacial aggregation, ensuring superior morphological and electrical durability.

**FIGURE 5:**
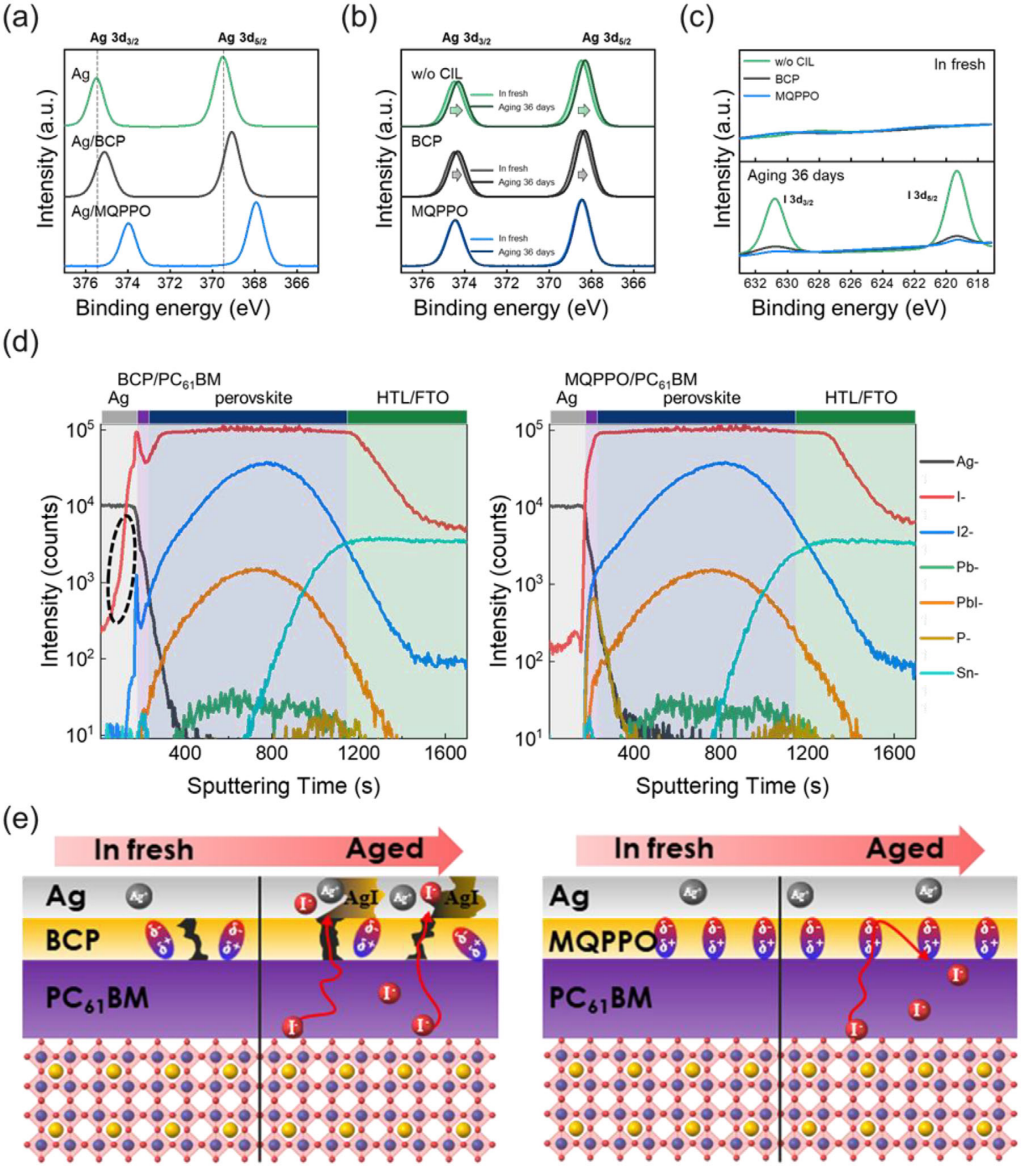
(a) Ag 3d XPS spectra of Ag and Ag/CILs. (b) Ag 3d and (c) I 3d XPS spectra of fresh and after 36 days storage FTO/Perovskite/PC_61_BM/CIL/Ag films with BCP or MQPPO layers. (d) ToF‐SIMS depth profiles of the PSCs after thermal aging at 110°C for 24 h. (e) Schematic illustration of the interface degradation and Ag corrosion protection mechanisms for PSCs with different CILs.

Subsequently, to investigate the interactions between Ag and the CIL, X‐ray photoelectron spectroscopy (XPS) measurements were conducted on Ag/CIL films (Figure 5a). For pristine Ag, the binding energies of Ag 3d_3/2_ and Ag 3d_5/2_ were observed at 375.5 and 369.5 eV, respectively. Compared to Ag/BCP, Ag/MQPPO exhibited a more noticeable shift of both peaks toward lower binding energies, indicating a stronger chemical interaction between MQPPO and Ag, which is also consistent with ESP results [64]. To further evaluate the long‐term stability of the Ag electrode, XPS measurements were carried out on fresh and aged (36 days) FTO/perovskite/PC_61_BM/CIL/Ag films. In all fresh films, Ag 3d peaks appeared at 368.48 and 374.48 eV, confirming the presence of metallic Ag^0^ with no detectable secondary species (Figure 5b; Table S4). After 36 days of aging, films without a CIL exhibited a binding energy shift to a 0.2 eV lower position, corresponding to the formation of insulating AgI caused by halide ion diffusion from the perovskite layer [14, 65]. In contrast, MQPPO‐based films exhibited negligible shifts in binding energy (<0.1 eV), indicating that MQPPO effectively suppresses halide penetration into the Ag electrode (Figure S16). The inhibition of halide diffusion was further corroborated by I 3d peaks analysis (Figure 5c). No iodide peaks were detected in the fresh films, whereas aged films without a CIL exhibited distinct I 3d peaks (619.33 and 630.78 eV), confirming AgI formation at the electrode. In contrast, MQPPO‐based samples showed the weakest iodide signal, which indicates effective suppression of AgI formation and supports improved long‐term operational stability of M‐PSCs [66]. Moreover, to investigate the halide migration behavior under accelerated conditions, the devices were subjected to thermal aging at 110°C for 24 h and characterized by time‐of‐flight secondary ion mass spectrometry (ToF‐SIMS). The analysis revealed a significant accumulation of iodide ions in the Ag electrode of the B‐PSC after thermal stress, whereas the M‐PSC exhibited minimal halide migration across the interface, as indicated with a black circle in Figure 5d. This confirms that MQPPO serves as an effective barrier against halide‐induced corrosion through robust chemical coordination with the Ag electrode [67]. The progression of electrode corrosion was further corroborated by the surface morphology of the devices (Figure S17). After 1,920 h of aging, severe corrosion was evident on the Ag cathode of the BCP‐based film, while the MQPPO‐based film exhibited no visible deterioration, maintaining its original metallic luster [68]. This exceptional stability is primarily attributed to the inhibition of AgI‐induced interfacial degradation through effective blocking of halide diffusion by MQPPO. This enabled M‐PSCs to preserve high recombination resistance and efficient charge extraction during long‐term storage in ambient, high‐moisture, and high temperature environments.

Based on these findings, the distinct role of MQPPO in stabilizing PSC operation is schematically summarized in Figure 5e. As evidenced by energy level change, BCP forms a relatively weak interfacial dipole, which is insufficient to effectively suppress charge recombination and limits photovoltaic performance [69]. Additionally, self‐aggregation of BCP molecules results in a non‐uniform interfacial coverage. Such morphological inhomogeneity accelerates perovskite degradation by moisture penetration and halide ion diffusion through the BCP/Ag interface [70]. Moreover, the relatively weak Ag─N interaction between BCP and Ag is insufficient to suppress the formation of insulating AgI, resulting in a substantial loss of device stability [71].

In contrast, the M‐PSCs achieve improved photovoltaic performance primarily owing to the energy‐level optimization driven by the strong interfacial dipole of MQPPO. Furthermore, their enhanced operational stability arises from two synergic mechanisms:

1. Inhibition of moisture penetration, mitigating perovskite decomposition
2. Formation of stronger Ag–N interactions, suppressing AgI formation

## 3 Conclusion

In summary, we demonstrated the successful implementation of MQPPO as a multifunctional CIL for high‐performance and stable p‐i‐n structured PSCs. With its strong and persistent interfacial dipole, MQPPO significantly enhances PV performance, achieving PCEs of 21.65% and 33.18% under 1‐sun and 1000 lux LED illumination, respectively. These improvements are attributed to reduced interfacial energy barriers, suppressed recombination, and more efficient charge extraction. Moreover, MQPPO substantially improves the long‐term operational device stability of PSCs, retaining over 80% of their initial efficiency after 1440 h, markedly outperforming B‐PSCs. The superior durability originates from the hydrophobic and morphologically stable interfacial layer formed by MQPPO, which effectively inhibits moisture ingress and halide ion diffusion. In addition, strong interfacial Ag─N coordination prevents the formation of insulating AgI by suppressing interfacial reactions between Ag and halide species. Collectively, these synergistic effects, dipole‐induced energy level alignment, interfacial morphological stability, and chemical passivation of the Ag electrode, enable MQPPO to serve as a multifunctional CIL that simultaneously delivers high efficiency and exceptional long‐term stability, underscoring its potential for practical and scalable perovskite photovoltaic applications.

## Conflicts of Interest

The authors declare no conflicts of interest.

## Supporting information




**Supporting File 1**: advs74802‐sup‐0001‐SuppMat.docx.

**Supporting File 2**: advs74802‐sup‐0002‐DataFile.zip.

## Data Availability

The data that support the findings of this study are available from the corresponding author upon reasonable request.
