# Supplementary material for: Reinforcement of Cathode Interface Using a Dipolar Small Molecule for Enhancing Operational Stability of Perovskite Solar Cells
Source: Adv Sci (Weinh). 2026 Mar 18;13(29):e24338. doi: 10.1002/advs.202524338 (PMC13205899; doi:10.1002/advs.202524338)
Supplement: Supplementary file 1 — Supporting File 1: advs74802‐sup‐0001‐SuppMat.docx. [file ADVS-13-e24338-s002.docx]

Supporting Information

**Reinforcement of Cathode Interface Using a Dipolar Small Molecule for Enhancing Operational Stability of**

**Perovskite Solar Cells**

*Dong Hyun Lee, Seok Woo Lee, Min Jun Choi, Ramesh Kumar Chitumalla, Sang Eun Yoon, Gyeong G. Jeon, Juan Anthony Prayogo, Dong Won Kim, Joonkyung Jang^*^, Jinhee Heo^*^, Dong Wook Chang^*^, Jong H. Kim^*^*

D. H. Lee, M. J. Choi, S. E. Yoon, G. G. Jeon, Dr. D. W. Kim, and Prof. J. H. Kim

Department of Molecular Science Technology, Ajou University, Suwon 16499, Republic of Korea

E-mail: jonghkim@ajou.ac.kr

S. W. Lee, J. A. Prayogo and Prof. D. W. Chang

Department of Energy and Chemical Materials Engineering and CECS Core Research Institute, Pukyong National University, 48547, Busan, Republic of Korea

E-mail: dwchang@pknu.ac.kr

R. K. Chitumalla, and Prof. J. Jang

Department of Nanoenergy Engineering, Pusan National University, Busan, 46241, Republic of Korea

E-mail: jkjang@pusan.ac.kr

Dr. J. Heo

Department of Materials Analysis, Korea Institute of Materials Science (KIMS), 51508, Changwon, Republic of Korea

E-mail: pidellis@kims.re.kr

Dr. D. W. Kim

AI-Superconvergence KIURI Translational Research Center, Ajou University, Suwon, Republic of Korea

**Keywords:** perovskite solar cells, organic small molecule, cathode interlayer, device stability, electrode corrosion

**Materials and Methods**

**Materials**

MQPPO was synthesized using previously reported methods.^[1]^ Lead(II) bromide (PbBr_2_) and lead(II) iodide (PbI_2_, 99.999%) were purchased from Alfa Aesar. [2-(3,6-Dimethoxy-9H-carbazol-9-yl)ethyl]phosphonic acid (MeO-2PACz) was purchased from TCI. Formamidinium iodide (FAI), methylammonium bromide (MABr), formamidinium bromide (FABr), propane-1,3-diammonium iodide (PDAI_2_), and phenethylammonium iodide (PEAI) were purchased from Greatcell Solar. Bathocuproine (BCP, 99.99%), 2-propanol (IPA, 99.5%), diethyl ether (DEE, 99.9%), N, N-dimethylformamide (DMF, 99.8%), dimethyl sulfoxide (DMSO, 99.9%), chlorobenzene (CB, 99%), ethanol (EtOH, 99.5%), and cesium iodide (CsI, 99.9%) were purchased from Sigma-Aldrich. [6,6]-phenyl-C61-butyric acid methyl ester (PC_61_BM) was purchased from 1-Material. C_60_ was purchased from Nano-C. The (CsI_0.05_((FAPbI_3_)_0.85_(MAPbBr_3_)_0.15_)_0.95_) precursor solution was prepared by dissolving 1.30 M FAI, 1.43 M PbI_2_, 1.30 M MABr, and 1.30 M PbBr_2_ using a co-solvent mixture of DMF and DMSO (8:3 v/v) totaling 1.0 ml; subsequently 57.89 µL of CsI (1.5 M in DMSO) was incorporated into the precursor solution with the solution stirred inside a N_2_-purged glove box. For the FA_0.825_Cs_0.175_Pb(Br_0.125_I_0.875_)_3_ perovskite precursor solution, FAI (187.0 mg), PbI_2_ (568.2 mg), FABr (13.6 mg), PbBr2 (79.8 mg), and CsI (65.9 mg) were in 1.0 mL of a DMF:DMSO (v/v = 9:1) co-solvent.

**Device fabrication**

***Fabrication of CsI_0.05_((FAPbI_3_)_0.85_(MAPbBr_3_)_0.15_)_0.95_-based PSCs:*** First, the glass/FTO substrates were sonicated in deionized water, acetone, and IPA for 15 minutes to clean them. After drying, UV-ozone treatment was applied to the substrates for 20 minutes to eliminate organic residues. MeO-2PACz (1 mg/mL in EtOH) was prepared by spin-coating at 3,000 rpm for 30 seconds onto the FTO-glass substrates. After spin-coating, the substrates were annealed at 100 °C for 10 minutes. Next, the perovskite solution was dropped and spin-coated onto the glass/FTO/MeO-2PACz via a consecutive two-step spin-coating process at 2,000 rpm for 5 seconds and 5,000 rpm for 15 seconds. During the second spin-coating step, the substrate was subjected to 1 ml diethyl ether drop casting and then annealed at 110 °C for 30 minutes. To create a passivation layer, PEAI (2 mg/mL in IPA) was spin-coated at 5,000 rpm for 30 seconds. After spin-coating the perovskite layers, the PC_61_BM (20 mg/mL in CB) layer was spin-coated at 1,500 rpm for 60 seconds. On the PC_61_BM layer, the BCP (0.5 mg/mL in ethanol) layer was spin-coated at 4,000 rpm for 30 seconds, while the MQPPO (1.5 mg/mL in IPA) layer was spin-coated at 4,000 rpm for 30 seconds. Thermal evaporation was used to deposit 100 nm thick silver electrodes at a vacuum pressure of 2.0×10⁻^6^ Torr, and the active area of the devices was measured at 0.105 cm².

***Fabrication of FA_0.825_Cs_0.175_Pb(Br_0.125_I_0.875_)_3_-based PSCs:*** The substrate cleaning and MeO-2PACz spin-coating processes were performed following the procedures described above. For the perovskite layer, the perovskite solution was spin-coated onto glass/FTO/MeO-2PACz at 3,000 rpm for 10 s. Immediately after coating, the substrate was transferred to a vacuum chamber for a 20 second vacuum-assisted crystallization process and then annealed at 100 °C for 20 min. Subsequently, PDAI_2_ solution (0.5 mg/mL in IPA) was spin-coated at 5,000 rpm for 30 seconds. The 15 nm C_60_ layer was thermally evaporated on the PDAI_2_ layer. Subsequently, the BCP, MQPPO, and silver layers were performed as mentioned above.

**Characterization**

A potentiostat (CompactStat, IVIUM) was used to measure the *J–V* curves of the PSC devices under standard AM 1.5G irradiation (100 mW cm⁻^2^) provided by a solar simulator (Newport, Oriel LCS-100, 94011A). The dependence of *J*_sc_ and *V*_oc_ on light intensity was evaluated by varying the input light using different optical density (OD) filters. The ideality factor can be evaluated from the average slope of the plot of *V*_oc_ versus light intensity (P_light_).

This equation calculates the light ideality factor (*n*):

$$n=\frac{q\Delta V_{\mathrm{oc}}}{KT\Delta(\ln(I))}$$

In this formula, *q* is the elementary charge, *K* is the Boltzmann constant, *I* stands for the light intensity, and *T* is the room temperature expressed in Kelvin.

An evaluation of the maximum power point tracking (MPPT) was conducted for the perovskite devices; a source meter (Keithley 2400) and the same solar simulator were employed.

Using LED lamps with calibration, measurements of *J–V* characteristics under low-intensity light were conducted using an illumination system (K3000, Mcscience) at lighting intensities of 400, 600, 800, and 1,000 lux with a sourcemeter (Keithley 4200). LED lamp illumination was quantified using a lux meter (GL Spectro lux meter, GL Optic).

Atomic force microscopy (AFM, XE-100, Park Systems) was applied to characterize the morphology of the perovskite films. Ultraviolet photoelectron spectroscopy (UPS) measurements were carried out using an X-ray photoelectron spectrometer (FC-XP10, Nexsa, Thermo Fisher Scientific). In contrast, X-ray photoelectron spectroscopy (XPS) measurements were performed using a different X-ray photoelectron spectrometer (FC-XP10, Nexsa, Thermo Fisher Scientific). FTIR spectra were measured by the FTIR spectrometer (Nicolet iS50, Thermo). ToF-SIMS was conducted on TOF-SIMS 5 (IONTOF) with a sputter energy of 1 keV.

Kelvin probe force microscopy (KPFM) measurements were carried out using an environment control AFM system (AFM 5300E, HITACHI) with an Rh-coated Si tip (SI-DF3-R) as the probe under halogen lamp conditions. A Si probe with a stiffness of 0.99 N/m and a resonance frequency of 24 kHz was used at 70% relative humidity (RH). The surface potential images have been attained under AC bias oscillating from – 2.5 V to +2.5 V with modulation frequency of 22.9 kHz for surface potential sensing. Contact angle measurements were performed on a contact angle analyzer (Phoenix 150, SEO).

Photoluminescence (PL) and time-resolved photoluminescence (TRPL) spectra of the perovskite films were measured using a spectrofluorometer (FS5 spectrofluorometer, Edinburgh instruments). TRPL measurement was performed using a 405 nm pulsed laser. The average lifetime (τ_ave_) was calculated using the following equation:

τ_ave_=(τ_1_^2^A_1_+τ_2_^2^A_2_)/(τ_1_A_1_+ τ_2_A_2_)

where τ_1_ and τ_2_ are the lifetimes, and A_1_ and A_2_ are the amplitudes of the respective components.^[2, 3]^

Transient photovoltage (TPV), transient photocurrent (TPC), and photo-induced charge extraction by linearly increasing voltage (Photo-CELIV) measurements were performed using a semiconductor parameter system (T4000, Mcscience). Photo-CELIV charge carrier mobility (*μ*) was calculated using the following equation:

$$\mu=\frac{2d^{2}}{3{(\Delta U/\Delta t) t}_{MAX}^{2}}$$

Where *(∆U/∆t)* is the voltage ramp of the applied triangular voltage pulse, *t^2^_MAX_* represents the duration at which the current density attains its peak value, and *𝑑* is the perovskite film thickness.^[4]^

Electrochemical impedance spectroscopy (EIS), intensity-modulated photovoltage spectroscopy (IMVS), and intensity-modulated photocurrent spectroscopy (IMPS) of the devices were measured using the potentiostat (CompactStat, IVIUM).

The IMPS and IMVS measurements were conducted using the same potentiostat, which was equipped with an LED system (IVIUM, IM1225).

The charge transport time (τ_ct_) is determined using the equation provided below:

τ_ct_ = 1/2πf_min_

f_min_ corresponds to the lowest current in the imaginary portion of the IMPS spectra within the low-frequency range.

The recombination time (τ_rec_) is determined using the equation provided below:

τ_rec_ = 1/2πf_min_

f_min_ corresponds to the lowest voltage in the imaginary portion of the IMVS spectra within the low-frequency range.^[5]^

**Computational details**

The electronic properties and molecular interactions of MQPPO were investigated using Density Functional Theory **(**DFT) calculations. All the DFT simulations were performed using the Gaussian 16 program.^[6]^ Geometry optimizations of MQPPO and BCP were performed without symmetry constraints using the B3LYP exchange–correlation functional in conjunction with the 6-31G(d) basis set.^[7]^ Vibrational frequency analyses were conducted at the same level of theory to ensure that all optimized structures correspond to true minima on the potential energy surface. Electrostatic potential surfaces (ESP) were generated based on the optimized molecular geometries to evaluate charge distribution and dipole polarization. Furthermore, to evaluate interfacial binding affinities, molecular interaction simulations were conducted using C_60_. The interaction energies between C_60_ and the CIL molecules (MQPPO or BCP) were calculated at the M06/6-31G(d) level.^[8]^ Basis set superposition error (BSSE) was corrected using the counterpoise method.^[9]^ The binding energy was evaluated as:

$$\boldsymbol{E}_{\boldsymbol{b}}\boldsymbol{=}\boldsymbol{E}_{\boldsymbol{complex}}\boldsymbol{-(}\boldsymbol{E}_{\boldsymbol{C}_{\boldsymbol{60}}}\boldsymbol{+}\boldsymbol{E}_{\boldsymbol{CIL}}\boldsymbol{)}$$

Interaction energies of MQPPO and BCP with Ag^+^ were calculated to assess chemical passivation ability. For Ag^+^, the LANL2DZ effective core potential (ECP) and corresponding basis set were employed, while the 6-31G(d) basis set was used for all other atoms. In addition, electron reorganization energies (EREs) were calculated based on Marcus theory^[10]^ by evaluating the energy differences between optimized neutral and anionic states to provide insight into the intrinsic electron-transfer properties of MQPPO and BCP.

**Supporting Note 1**

**Ambient Stability:** Unencapsulated devices were stored at 25 °C and 40% RH in dark. For comparison, stability was also evaluated under (i) 1-sun (AM 1.5G, 100 mW cm^-2^) and (ii) 1,000 lux LED illumination. Shelf stability tests were conducted to assess long-term device durability.

**Moisture Stability:** Unencapsulated devices were aged at 25 °C and 70% RH in dark. Shelf stability of the devices was evaluated to investigate the moisture-induced degradation.

**Accelerated Thermal Stability:** Thermal robustness was evaluated using maximum power point tracking (MPPT) at 85 °C and 40% RH with unencapsulated devices. The unencapsulated devices were continuously monitored at the maximum power point (MPP) under constant illumination (AM 1.5G, 100 mW cm⁻²) to record operational decay.

**
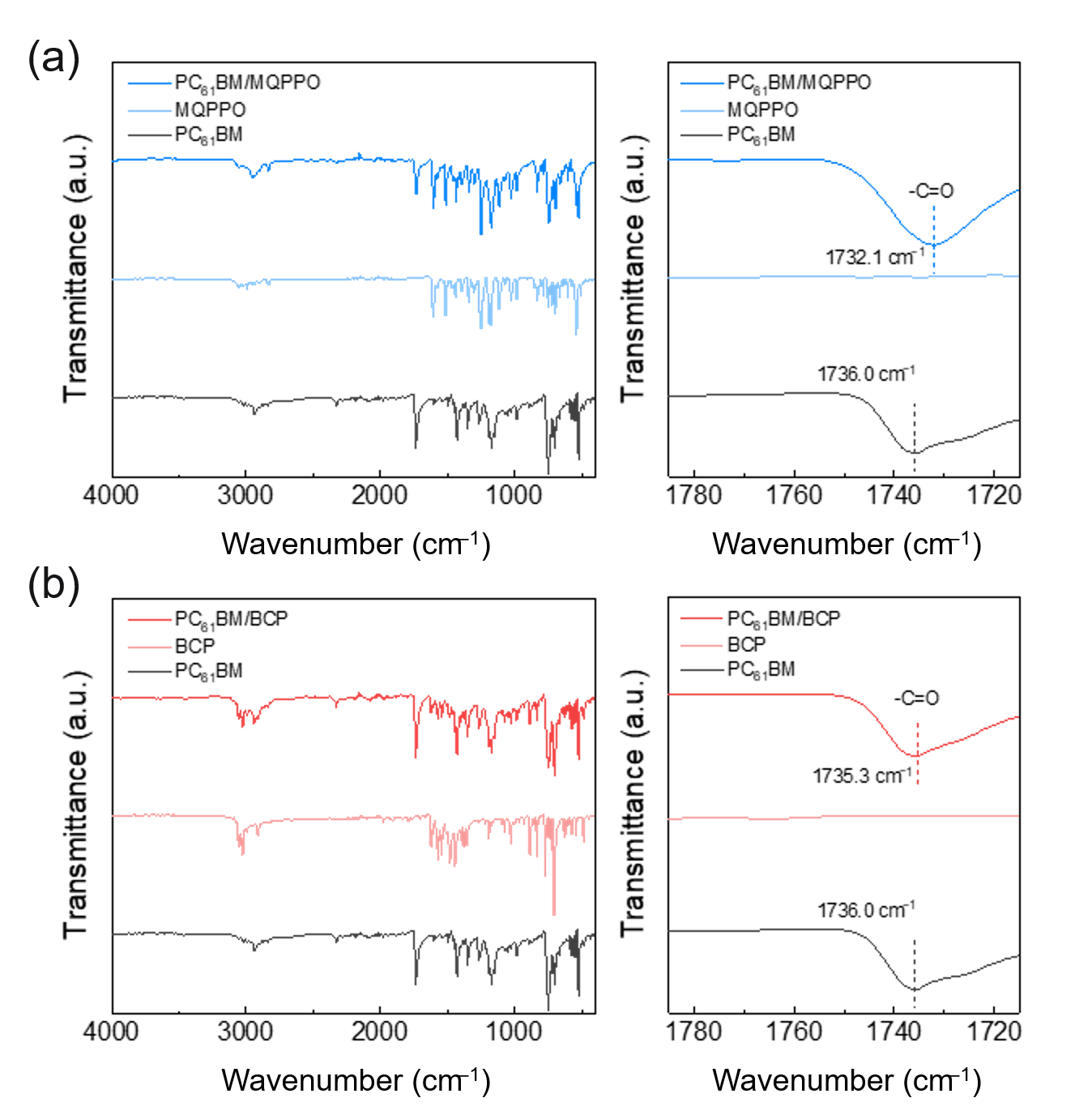
**

**Figure S1**. (a) FTIR spectra of the pristine PC_61_BM, MQPPO, and PC_61_BM/MQPPO blend. (b) FTIR spectra of the pristine PC_61_BM, BCP, and PC_61_BM/BCP blend.


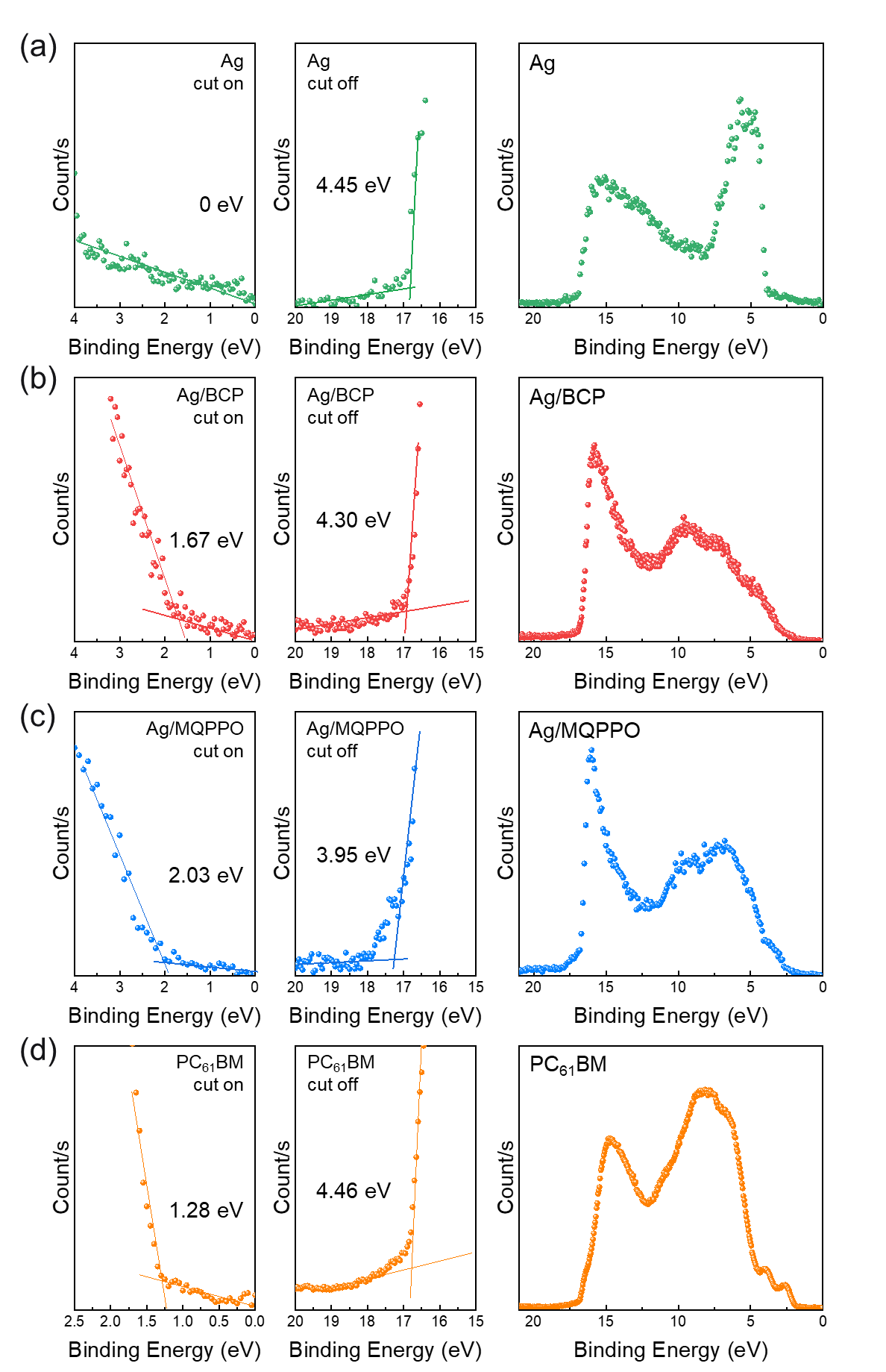


**Figure S2.** UPS spectra of (a) pristine Ag, (b) Ag/BCP, and (c) Ag/MQPPO (d) PC_61_BM layer.


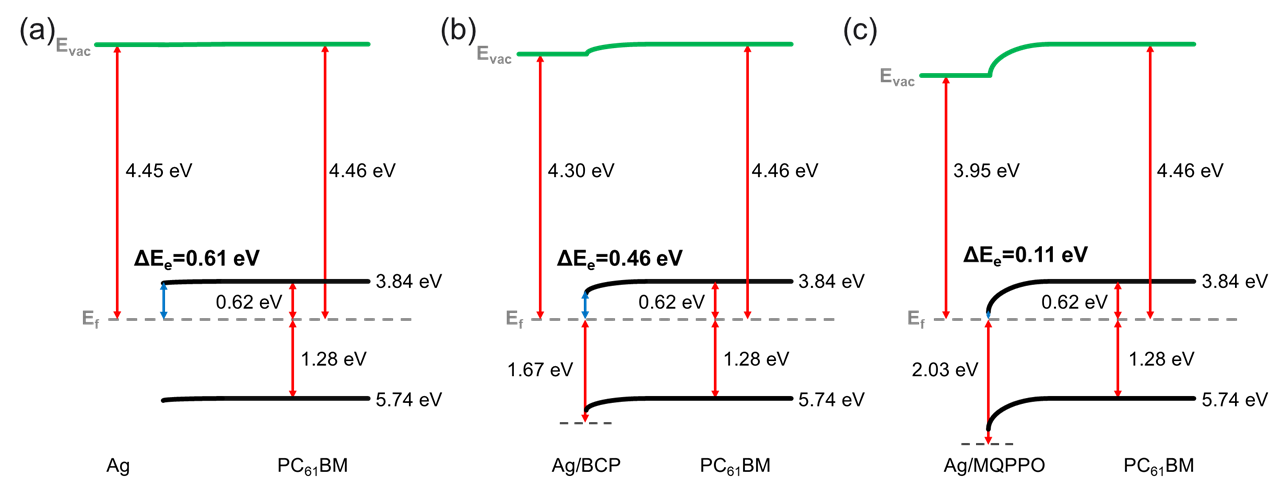


**Figure S3.** Energy-level diagrams of (a) PC_61_BM, (b) Ag/BCP/PC_61_BM and (c) Ag/MQPPO/PC_61_BM interfaces. The vacuum level shift and the electron extraction barrier (ΔE_e_) are indicated for each system.

**
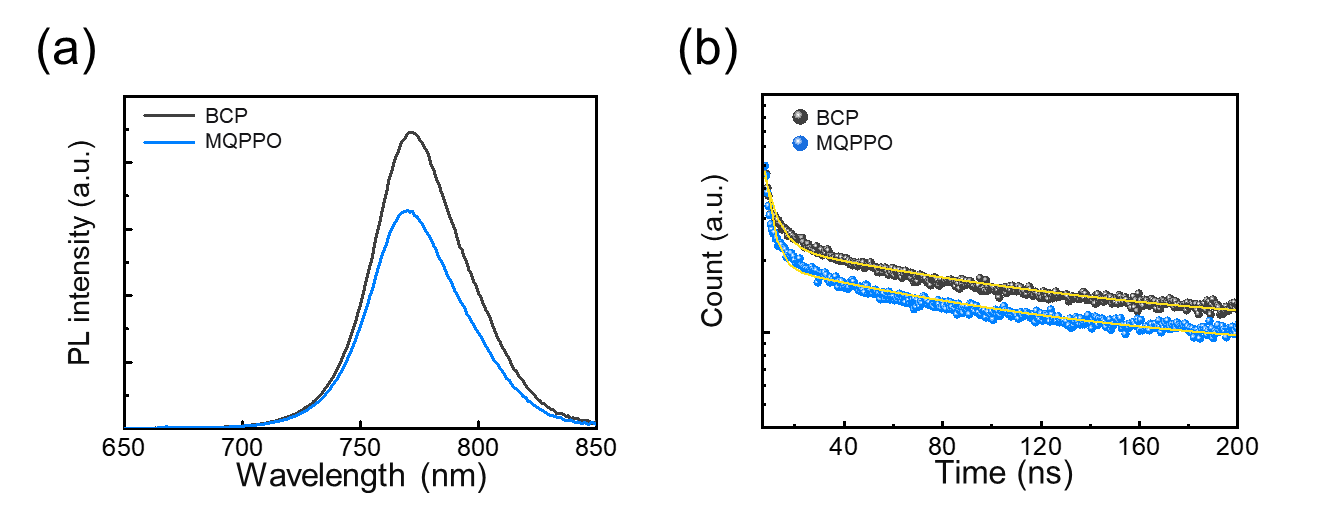
**

**Figure S4.** (a) Steady-state and (b) time-resolved photoluminescence (PL) spectra of the perovskite/PC_61_BM films with CILs.


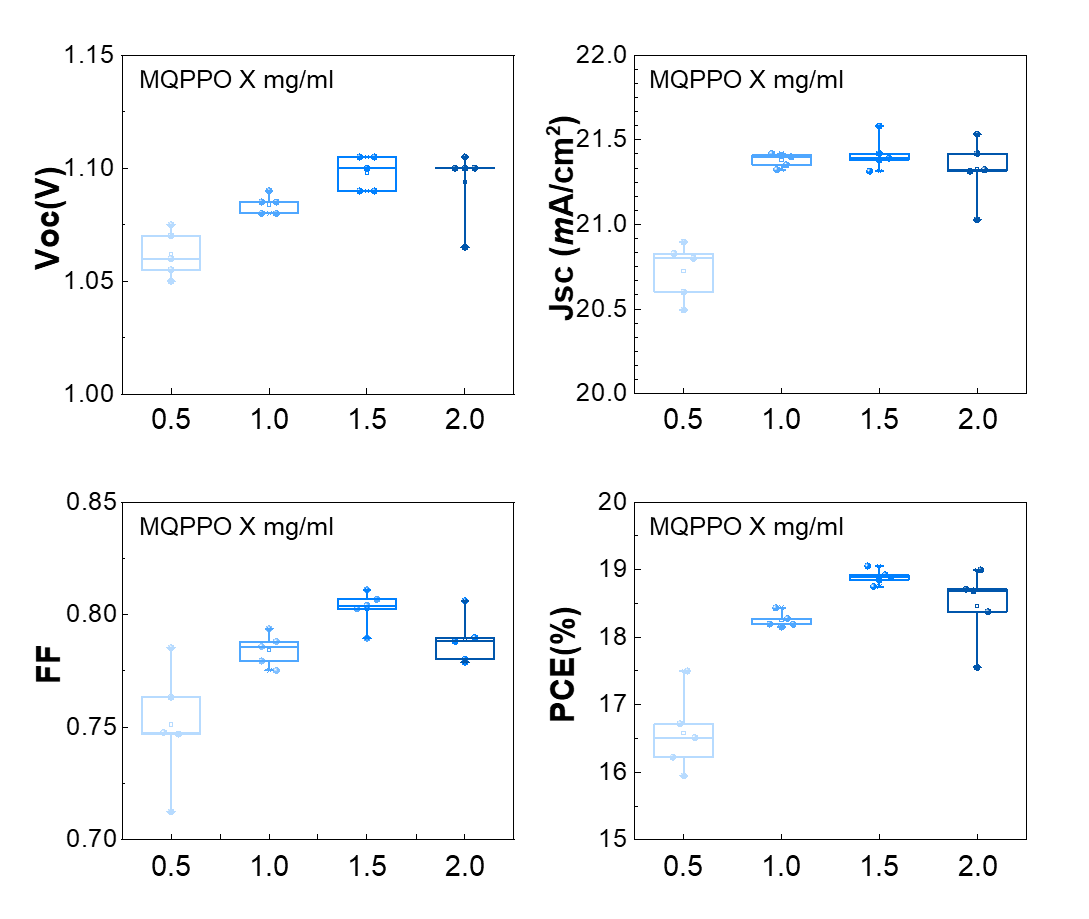


**Figure S5.** Optimization of MQPPO concentration for cathode interlayer. Statistical distribution of photovoltaic parameters, including *V*_OC_, *J*_SC,_ FF, and PCE, for p-i-n perovskite solar cells fabricated with different MQPPO concentrations (0.5, 1.0, 1.5, and 2.0 mg/mL) in IPA.


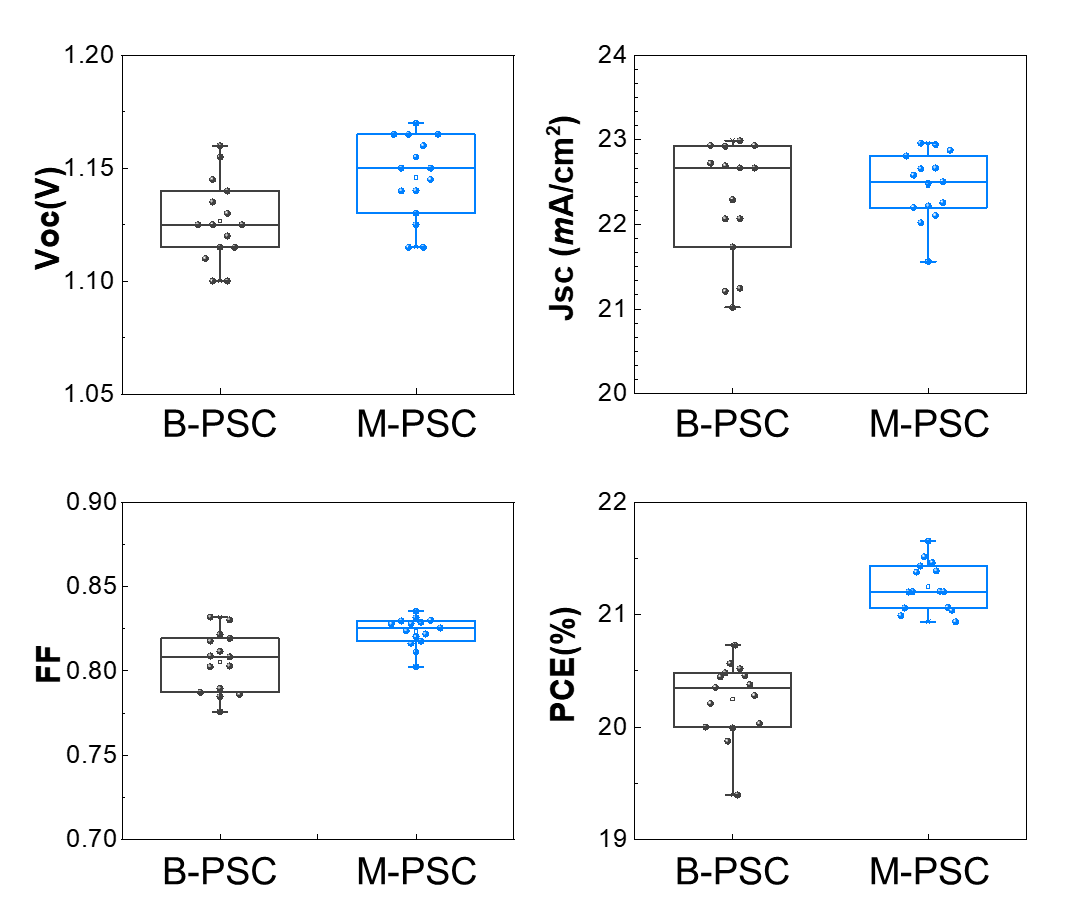


**Figure S6.** Distributions of photovoltaic parameters for PSCs with different CILs, based on 15 devices for each.


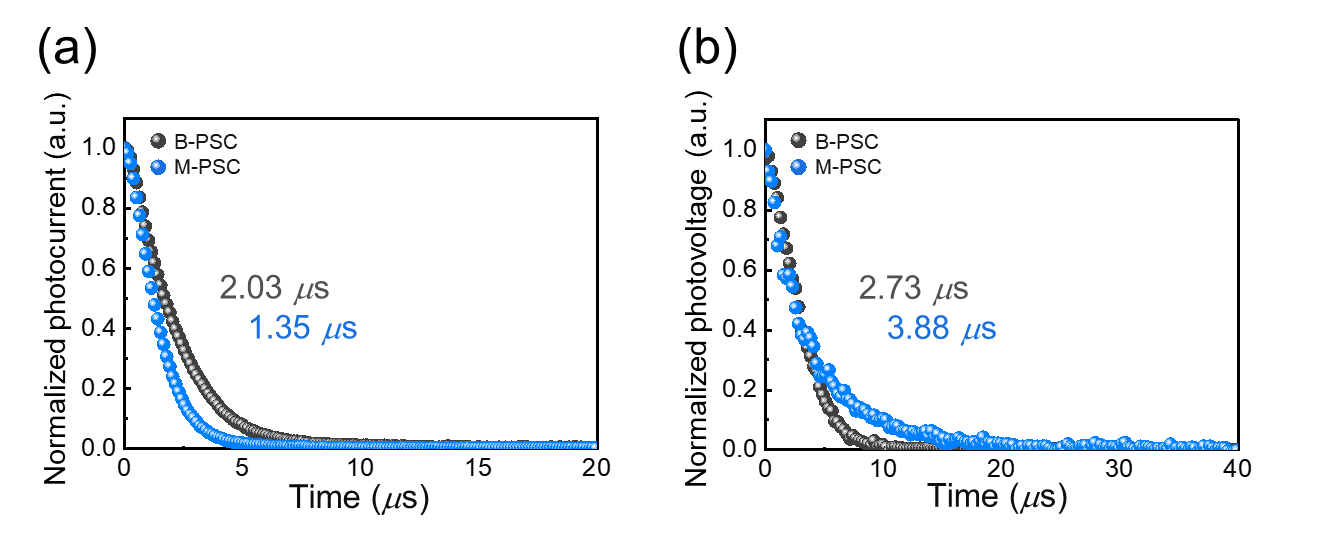


**Figure S7.** (a) Transient photocurrent (TPC), (b) transient photovoltage (TPV) decay curves with different CILs.


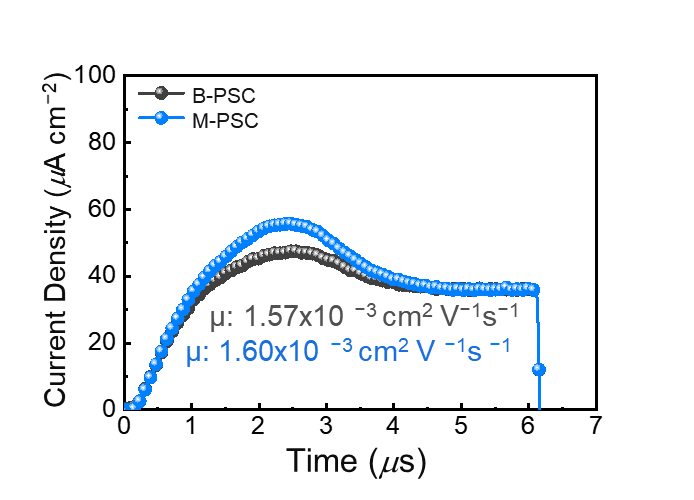


**Figure S8.** Photo-CELIV curves with different CILs.


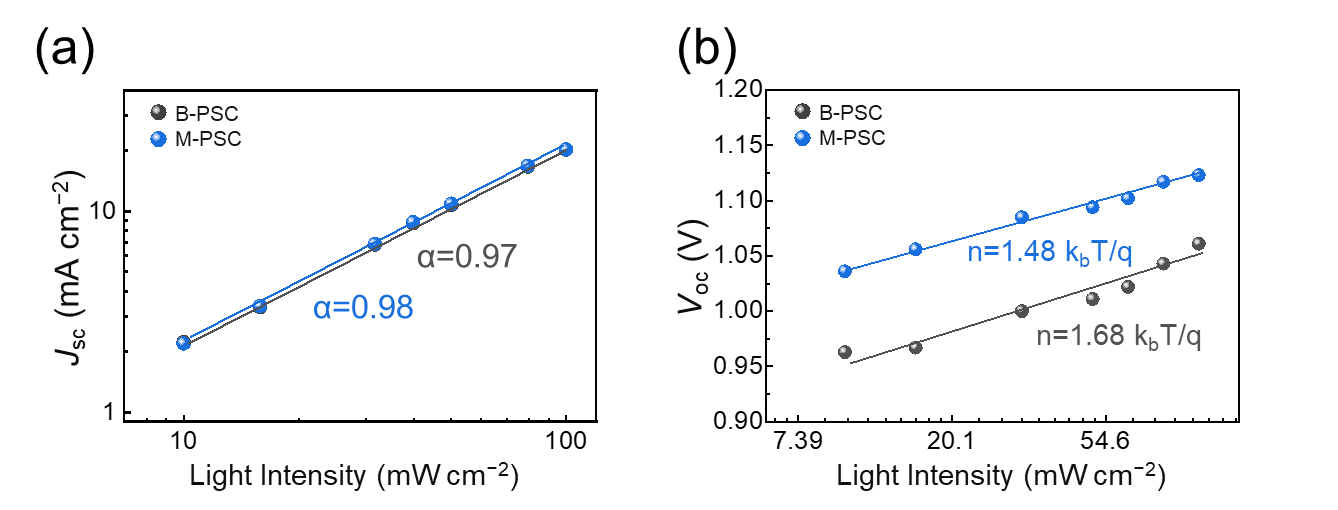


**Figure S9.** Light intensity dependence of (a) *J*_SC_ and (b) *V*_OC_ for PSCs with different CILs.


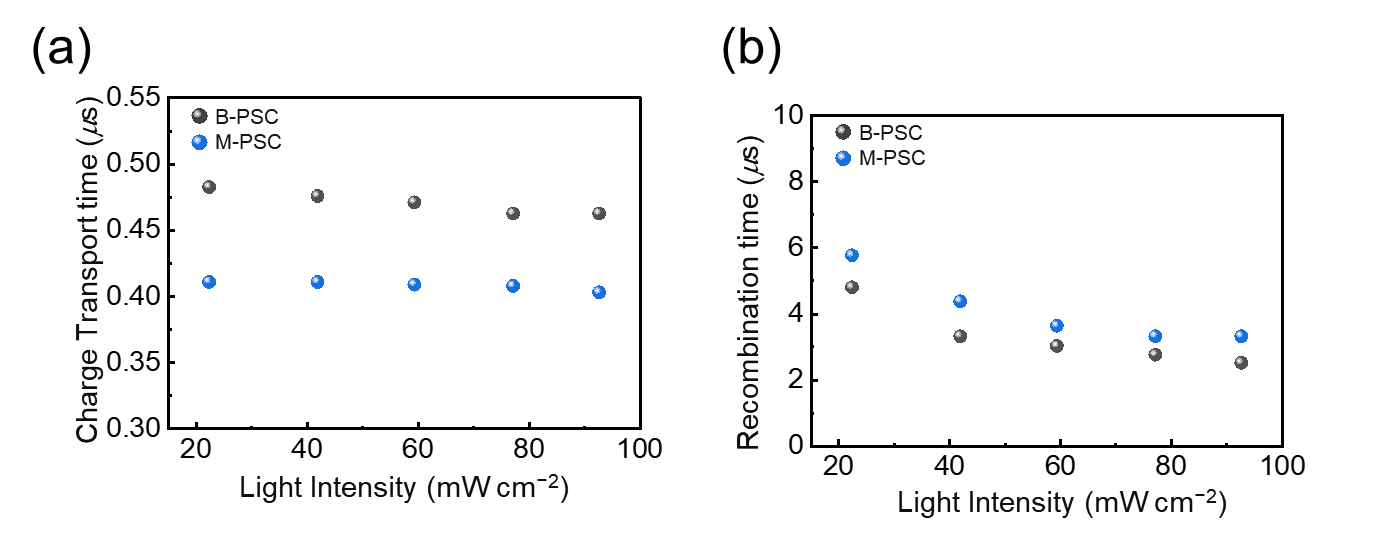


**Figure S10.** (a) Charge transport time and (b) charge recombination time dependent on light intensity for PSCs with different CILs.

**
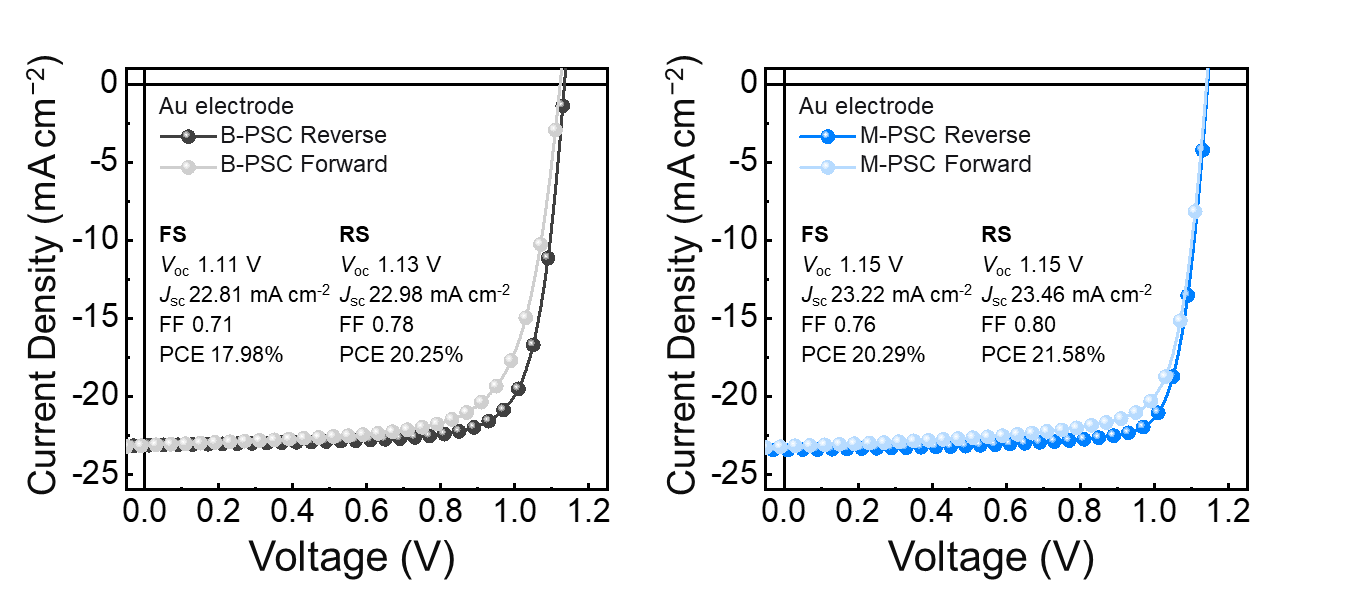
**

**Figure S11.** *J–V* curves for B-PSC and M-PSC using an Au electrode under AM1.5G 1-sun illumination.


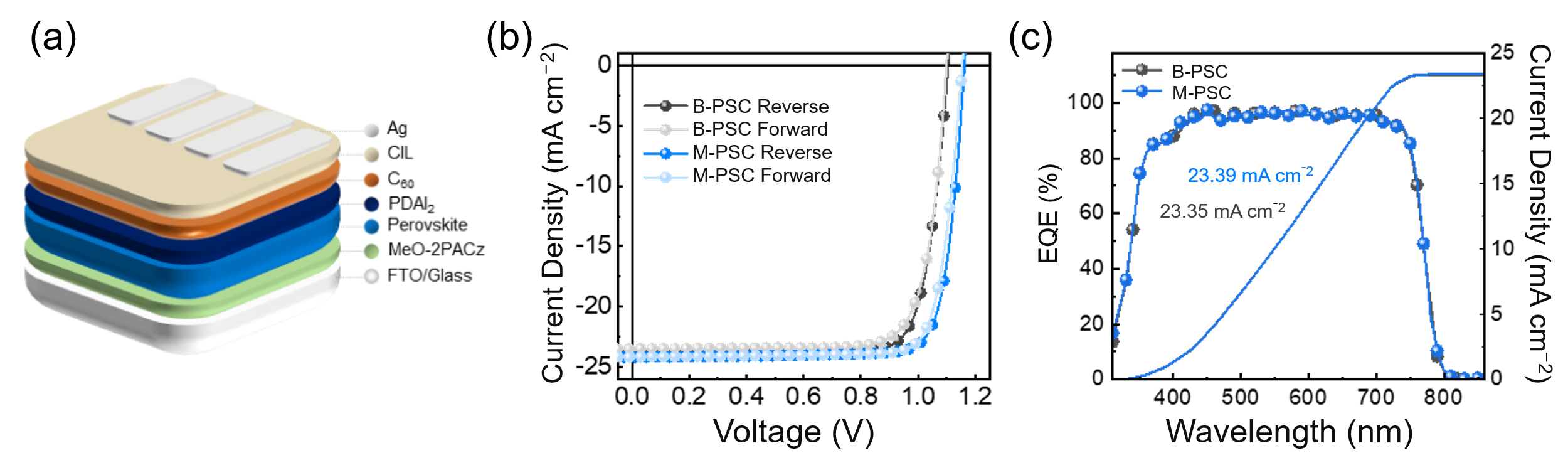
 **Figure S12.** Photovoltaic performance of PSCs using a different perovskite composition (FA_0.825_Cs_0.175_Pb(Br_0.125_I_0.875_)_3_). (a) Schematic illustration of the device architecture. (b) *J*–*V* characteristics of the B-PSC and M-PSC devices. (c) EQE spectra and corresponding integrated *J*_SC_.

**
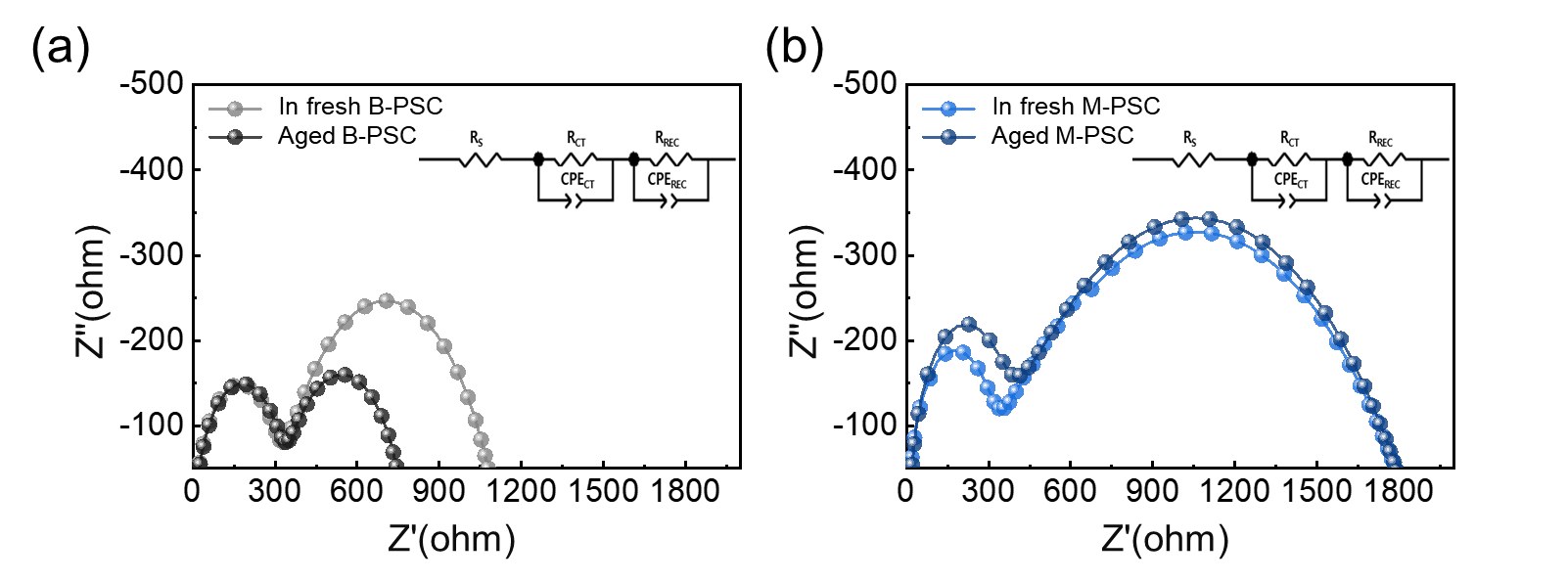
**

**Figure S13.** Nyquist plot for fresh PSCs and after 24 hours of storage based on (a) BCP and (b) MQPPO.


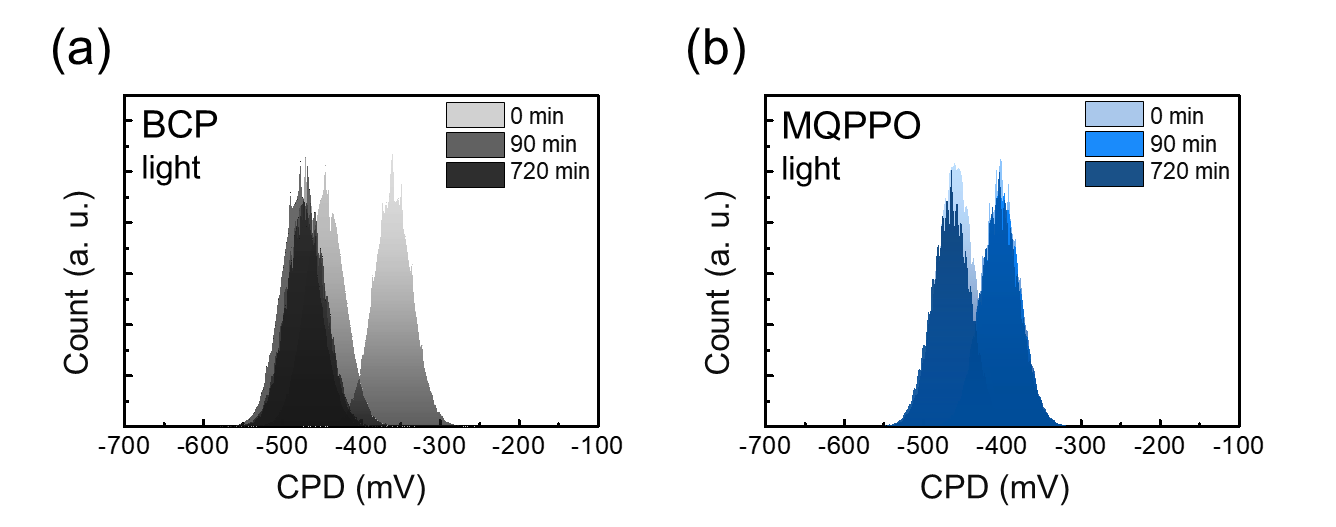


**Figure S14.** CPD counts distribution in light illumination conditions, obtained from each CPD map for the PSCs based on (a) BCP and (b) MQPPO.


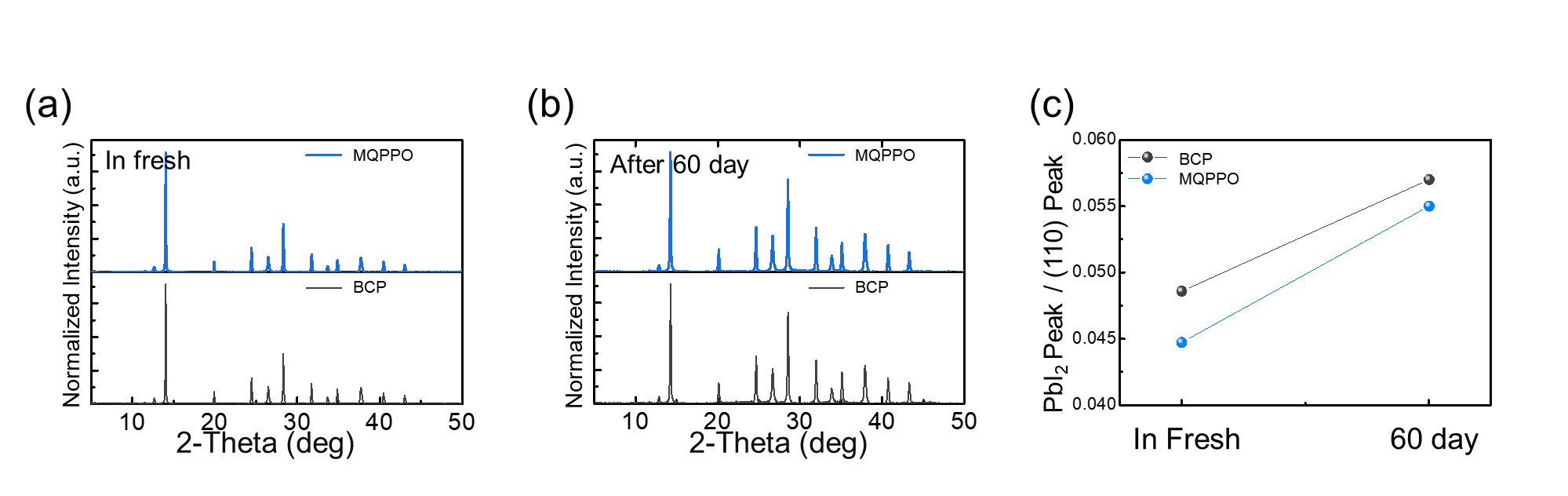


**Figure S15.** The X-ray diffraction (XRD) pattern of (a) fresh perovskite films and (b) perovskite films after 60 days of storage. (c) The molar ratio between PbI_2_ XRD peak and perovskite (110) XRD peak.


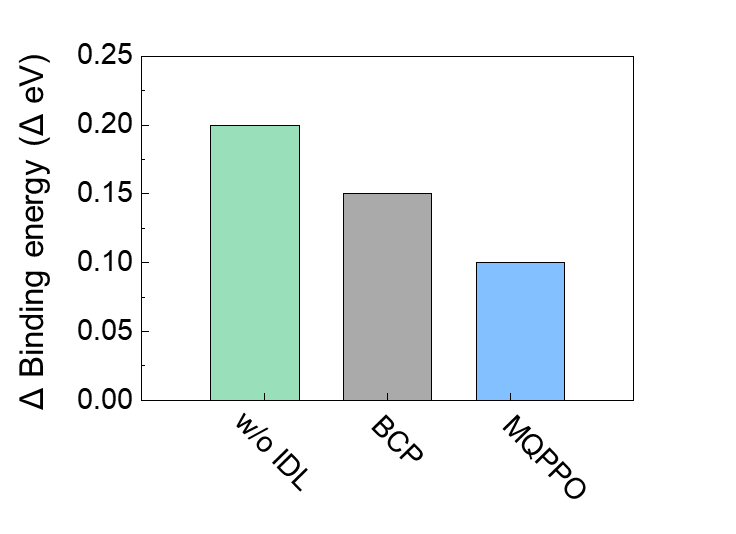


**Figure S16.** Ag 3d XPS binding energy shift diagram between fresh and after 36 days of storage film.
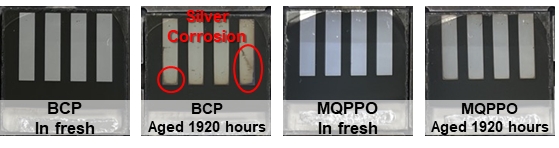


**Figure S17.** Photographs of PSCs in both fresh states and after 1,920 h of aging under dark conditions.

**Table S1.** Detailed time-resolved PL parameters of perovskite films with different CILs. The average lifetime was calculated from τ_ave_ = (τ_1_^2^A_1_ + τ_2_^2^A_2_)/(τ_1_A_1_ + τ_2_A_2_).

| **Condition** | **Bi-exponential fit** | | **Decay Amplitude ratio** | | **Average** |
| --- | --- | --- | --- | --- | --- |
|  | **τ_1_ [ns]** | **τ_2_ [ns]** | **A_1_ [%]** | **A_2_ [%]** | **τ_ave_ [ns]** |
| **BCP** | 4.90 | 117.84 | 88.2% | 4.0% | 63.77 |
| **MQPPO** | 3.12 | 109.17 | 96.6% | 2.8% | 56.32 |

**Table S2.** Performance parameters of the PSCs based on (FA_0.825_Cs_0.175_Pb(Br_0.125_I_0.875_)_3_) using various CILs under 1-sun illumination.

| **Devices^a^** | **Scan**  **direction** | ***V*_OC_**  **[V]** | ***J*_SC_ [mA cm⁻^2^]** | **FF** | **PCE  [%]** |
| --- | --- | --- | --- | --- | --- |
| B-PSC | FS | 1.11 | 23.46 | 0.80 | 20.64 |
|  | RS | 1.10  (1.12) | 24.00  (23.68) | 0.80  (0.78) | 21.31  (20.72) |
| M-PSC | FS | 1.16 | 24.14 | 0.82 | 20.80 |
|  | RS | 1.17  (1.17) | 24.16  (23.81) | 0.82  (0.80) | 23.18  (22.36) |

^a^The average photovoltaic parameters of PSCs were obtained for 10 devices

**Table S3.** The EIS fitting parameters for fresh PSCs and after 24 hours storage PSCs with different CILs.

| **Condition** | **Parameters** | **In Fresh** | **Aging 24hr** | **Deviation (%)** |
| --- | --- | --- | --- | --- |
| **B-PSC** | **R_s_ [Ω]** | 10.68 | 11.21 | 4.963 |
|  | **R_CT_ [Ω]** | 300.2 | 320.9 | 6.895 |
|  | **R_REC_ [Ω]** | 799.9 | 439.7 | 45.03 |
| **M-PSC** | **R_s_ [Ω]** | 6.950 | 7.010 | 0.863 |
|  | **R_CT_ [Ω]** | 270.4 | 277.4 | 2.589 |
|  | **R_REC_ [Ω]** | 1564 | 1557 | 0.448 |

**Table S4.** XPS Ag 3d_5/2_ and 3d_3/2_ peak for fresh and after 36 days storage FTO/Perovskite/PC_61_BM/Ag and FTO/Perovskite/PC_61_BM/CIL/Ag films.

| **Condition** | **In Fresh** | | **After 36 days** | |
| --- | --- | --- | --- | --- |
|  | **Ag 3d_5/2_ [eV]** | **Ag 3d_3/2_ [eV]** | **Ag 3d_5/2_ [eV]** | **Ag 3d_3/2_ [eV]** |
| **FTO/Perovskite/PC_61_BM/Ag** | 368.48 | 374.48 | 368.3 | 374.28 |
| **FTO/Perovskite/PC_61_BM/BCP/Ag** | 368.48 | 374.48 | 368.3 | 374.33 |
| **FTO/Perovskite/PC_61_BM/MQPPO/Ag** | 368.48 | 374.48 | 368.4 | 374.43 |

**References**

[1] S. W. Lee, X. Fan, D. R. Whang, J. W. Jang, H. Choi, D. W. Chang, B. R. Lee, *J. Inf. Disp.*, **2023**, *24*, 189–198.

[2] J. Dai, J. Xiong, N. Liu, Z. He, Y. Zhang, S. Zhan, B. Fan, W. Liu, X. Huang, X. Hu, D. Wang, Y. Huang, Z. Zhang, J. Zhang, *Chem. Eng. J.*, **2023**, *453*, 139988.

[3] Y. Liu, B. J. Kim, H. Wu, L. Yuan, H. Zhu, A. Liu, E. M. J. Johansson, *ACS Appl. Energy Mater.*, **2020**, *3*, 9817–9823

[4] J. Peng, Y. Chen, K. Zheng, T. Pullerits, Z. Liang, *Chem. Soc. Rev.*, **2017**, *46*, 5714–5729.

[5] Z. Zhang, J. Liu, H. Bi, L. Wang, Q. Shen, S. Hayase, *Chem. Eng. J.*, **2024**, *483*, 149345.

[6] M. J. Frisch, G. W. Trucks, H. B. Schlegel, G. E. Scuseria, M. A. Robb, J. R. Cheeseman, G. Scalmani, V. Barone, G. A. Petersson, H. Nakatsuji, X. Li, M. Caricato, A. V. Marenich, J. Bloino, B. G. Janesko, R. Gomperts, B. Mennucci, H. P. Hratchian, J. V. Ortiz, A. F. Izmaylov, J. L. Sonnenberg, D. Williams-Young, F. Ding, F. Lipparini, F. Egidi, J. Goings, B. Peng, A. Petrone, T. Henderson, et al., Gaussian 16, Revision C. 02, Gaussian, Inc., Wallingford CT, **2019**.

[7] A. D. Becke, *J. Chem. Phys.*, **1993**, 98, 5648.

[8] Y. Zhao D. G. Truhlar, *Theor. Chem. Acc.*, **2008**, 120, 215.

[9] S. Simon, M. Duran, J. J. Dannenberg, *J. Chem. Phys*., **1996**, 105, 11024.

[10] R. A. Marcus, *Theor. Chem. Acc.*, **1956**, 24, 966.
